# Endocrine Disruptors and Obesity: An Examination of Selected Persistent Organic Pollutants in the NHANES 1999–2002 Data

**DOI:** 10.3390/ijerph7072988

**Published:** 2010-07-23

**Authors:** Mai A. Elobeid, David W. Brock, David B. Allison, Miguel A. Padilla, Douglas M. Ruden

**Affiliations:** 1 Department of Biostatistics, University of Alabama at Birmingham, 1665 University Boulevard, Birmingham, AL, USA, 35294; E-Mail: maielobeid@gmail.com; 2 Division of Cardiovascular Disease, Department of Medicine, University of Alabama at Birmingham, 1665 University Boulevard, Birmingham, AL 35294, USA; E-Mail: David.Brock@uvm.edu; 3 Department of Nutrition Sciences, University of Alabama at Birmingham, 1665 University Boulevard, Birmingham, AL 35294, USA; 4 Clinical Nutrition Research Center, University of Alabama at Birmingham, 1665 University Boulevard, Birmingham, AL 35294, USA; 5 Department of Psychology, Old Dominion University, 250 Mills Godwin Building, Norfolk, VA 23529, USA; E-Mail: mapadill@odu.edu; 6 Department of Mathematics and Statistics, Old Dominion University, 250 Mills Godwin Building, Norfolk, VA 23529, USA; 7 Institute of Environmental Health Sciences, Wayne State University, 259 Mack Avenue, Detroit, MI 48201, USA; E-Mail: douglasr@wayne.edu

**Keywords:** Obesity, endocrine disruptors, waist circumference, persistent organic pollutants, public health

## Abstract

Recent evidence suggests that endocrine disrupting chemicals (EDCs) may cause perturbations in endogenous hormonal regulation that predispose to weight gain. Using data from NHANES (1999–2002), we investigated the association between body mass index (BMI), waist circumference (WC) and selected persistent organic pollutants (POPs) via multiple linear regressions. Consistent interaction was found between gender, ln oxychlordane and ln p,p’ DDT. Also, we found an association between WC and ln oxychlordane and ln hpcdd in subjects with detectable levels of POPs, whereas an association between WC and ln p,p’ DDT was observed in all subjects. Furthermore, ln Ocdd showed an increase with higher WC and BMI, whereas, ln trans-nonachlor decreased with higher BMI. Hence, BMI and WC are associated with POPs levels, making the chemicals plausible contributors to the obesity epidemic.

## 1. Introduction

The prevalence of obesity has risen substantially in both adults and children, and is recognized as a serious public health problem [1]. Beyond body mass index (BMI) Kg/m^2^, which is the most commonly used proxy index of obesity, indicators of regional fat distribution such as waist circumference (WC) have been linked to cardiovascular disease [2–6], insulin resistance syndrome [7–10], and increased risks of breast, colorectal, and renal cancer [11–13]. A recent study showed small but consistent increases in WC in the U.S. population over calendar time from the 1960s to the present even when measured at any fixed BMI level [14].

While much has been written about the reasons behind the global obesity epidemic, industrial toxicants found in the environment, the food system, and humans have just begun to receive significant attention. Recent evidence suggests that environmental contaminants known or presumed to disrupt endocrine systems, appropriately termed endocrine disrupting chemicals (EDCs), may play a role in the growing problem of obesity [15,16]. These contaminants are mostly persistent in the environment and are known as persistent organic pollutants (POPs). It is plausible that POPs lead to preferential increases in abdominal fat because it is known that the hormonal milieu has profound effects on the anatomic distribution of fat. However, whether associations between lifetime exposure to low doses of a mixture of various POPs and measures of adiposity exist in the general population is not known. Given that almost all persons are exposed to POPs, the public health importance of a relation of these chemicals with weight gain may be substantial, despite a relatively modest association with any individual POPs.

Recently, a dose-response relation was observed between serum concentrations of POPs and metabolic syndrome [17], insulin resistance [18], and diabetes [19]. Surprisingly, in people with undetectable levels of POPs, the typically robust association between obesity and diabetes was not observed [19]. However, an unanswered question is whether POPs might directly contribute to obesity in the population. Lee and colleagues have offered some useful evidence, but their study only treated BMI and WC as covariates, not as outcomes to be modeled. Hence, to explore this possibility of POPs’ associations with adiposity indicators, we investigated the associations among BMI, WC and selected POPs,-which do not have high affinities for the Aryl hydrocarbon receptor (Ahr)-, in 1999–2002 data from the National Health and Nutrition Examination Survey (NHANES 99-02). Some of the examined POPs are dioxins, which are a family of chlorinated hydrocarbon compounds known chemically as dibenzo-p-dioxins. Dioxins are highly toxic and persist in the environment for extended periods. They are produced during incineration of wastes and are produced as contaminants in chemical manufacturing processes [20]. We examined the measured POPs in that dataset which are 1,2,3,4,6,7,8-heptachlorodibenzo-*p*-dioxin (hpcdd), 1,2,3,4,6,7,8,9-octachlorodibenzo-*p*-dioxin (Ocdd), oxychlordane, *trans*-nonchlor, and *p,p*′-DDT. The POPs that have higher affinities for the Ahr in the NHANES dataset, such as 2,3,7,8-tetrachlorodibenzo-p-dioxin (TCDD), did not show significant associations with either BMI or WC.

## 2. Experimental Section

### 2.1. Sample

We used data from the publicly available nationally representative cross-sectional surveys of the U.S. non-institutionalized civilian population conducted by the US National Center for Health and Statistics (NCHS), (*NHANES 1999–2000, 2001–2002).* These surveys have the same basic structure and plan, and they both contain data on age, gender, race, height, weight, and WC.

### 2.2. Measures

Anthropometric measures including height, weight, and WC were obtained via standardized protocols as described elsewhere (http://www.cdc.gov/nchs/data/nhanes/frequency/bmxdoc.pdf).

Measures of polychlorinated dibenzo-*p*-dioxins (PCDDs), dobenzofurans (PCDFs), and non-ortho substituted or coplanar polychlorinated biphenyls (cPCBs), other polychlorinated biphenyls (PCBs), persistent chlorinated metabolites were assessed in participants on a one-third serum samples. For this analysis, data from the two surveys (1999–2000 and 2001–2002) were aggregated. The NHANES standardized home interview was followed by a detailed physical examination in a mobile evaluation clinic or the participant’s home. PCDDs, PCDFs, PCBs, and organochlorine pesticides were all measured in serum samples as individual chemicals by high resolution gas chromatography mass spectrometry (GC–MS) using isotope dilution for quantification. The POPs were provided by NHANES and adjusted for serum total cholesterol and triglycerides. All POPs levels were transformed by taking the natural log (ln) of each in order to improve linearity and analyzed in their transformed form.

We selected five POPs (present in 80% of the NHANES population): 1,2,3,4,6,7,8-heptachlorodibenzo-*p*-dioxin (hpcdd); 1,2,3,4,6,7,8,9-octachlorodibenzo-*p*-dioxin (Ocdd); oxychlordane; *trans*-nonchlor, and p,p′-DDT. Based on the above criteria, a total of 2,464 participants with valid BMI scores and 2,448 participants with valid WC score were examined.

### 2.3. Statistical Analysis

The primary analysis used three regression models to test all available participants for associations with overall adiposity as assessed by BMI and WC. Two models regressed BMI and WC on gender, ethnicity, age, age squared, and POPs. The third model regressed WC on the previous predictors while controlling for BMI. We will refer to these models as additive models. Each of these models was extended by including two-way interactions between gender and the POPs. Secondary/sensitivity analyses employed all of the same models as the primary analyses but evaluated only participants with detectable levels of POPs. All parameter interpretations will be done on using standard deviation units based on standardized regression parameters; *b_j_ = β̂_j_* *S_j_* / *S_y_*.

All analyses, including descriptive statistics, were conducted using SAS-Callable SUDDAN 9.0.1, which estimates standard errors using the sampling weights, strata, and primary sampling units (PSU) from NHANES taking into account for the complex sampling procedures used. For details on the sampling procedures used, visit the NHANES website at http://www.cdc.gov/nchs/nhanes.htm.

## 3. Results and Discussion

### 3.1. Descriptive Statistics

Descriptive statistics for gender, ethnicity, age, BMI, and WC are displayed in Table 1.

### 3.2. Primary Analysis

#### 3.2.1. Additive Models

Results of the additive regression models for all participants are presented in Table 2. In the additive BMI model there are significant main associations for ethnicity (p = 0.0466), age (p < 0.00005), age squared (p < 0.00005), ln Ocdd (p = 0.0099), and ln DDT (p = 0.0353), with the overall model accounting for 8% of the BMI variance. Natural log (ln) values for the POPs were used, as is typical in this type of study [19]. Expressed in standard deviation (SD) units, each 1 SD increase in ln Ocdd, BMI is associated with an increase of 0.249 BMI units and every SD unit increase in ln DDT is associated with a BMI increase of 0.106 units (*i.e*., the Beta value for ln DDT = 0.48; Table 2). The joint test p-value for the additive BMI model, which is the simultaneous test of all 5 POPs, is 0.0001. Additionally, the POPs accounted for 2% of the BMI variance.

For the additive WC model, there are significant associations for gender (p < 0.00005), age (p < 0.00005), age squared (p < 0.00005), and ln Ocdd (p = 0.0052). The model accounted for 16% of the variance in WC. WC increases by 1.76 centimeters with each SD unit increase in ln Ocdd. The joint test p-value for the additive WC model is 0.0008.

For testing the additive WC model controlling for BMI, there was a significant association for gender (p < 0.00005) and ln hpcdd (p = 0.0091) with the model accounting for 88% of the WC variance (Table 2). In this model, males tended to have 6.40 centimeters higher WC than females. However, WC decreased by 0.163 centimeters for every SD unit increase in ln hpcdd. This is an exception to the general observation that WC increases with POPs exposure. The joint test p-value for testing the POPs simultaneously for the additive WC model controlling for BMI is 0.0798, which is nearly statistically significant.

#### 3.2.2. Interaction Models

Table 3 displays the results of the interaction regression models for all participants, and Table 4 displays the interaction regression equations for males and females. The interaction BMI model accounted for 10% of the variance and showed significant associations for ln Ocdd (p = 0.0091) and ln DDT (p = 0.0020) (Table 3). The model also revealed significant gender interactions with ln hpcdd (p = 0.0033) and ln DDT (p = 0.0060) (Table 3). Ln Ocdd was positively associated with BMI increasing by 0.541 for every SD unit increase in ln Ocdd (Table 3).

For the ln hpcdd interaction, male BMIs increase by 0.413 and female BMIs decrease by 1.71 for every SD unit increase in ln hpcdd (Table 4). However, for the ln DDT interaction, male BMI decreases by 0.050 and female BMI increases by 0.344 for every SD unit increase of ln DDT (Table 4). The joint test p-value for simultaneously testing the interaction for the interaction BMI model is 0.0005.

The interaction WC model accounted for 18% of the WC variance and showed significant associations for ln Ocdd (p = 0.0120) and ln DDT (p = 0.0010) (Table 3). The model also showed significant gender interactions with ln hpcdd (p = 0.0059) and ln DDT (p = 0.0003) (Table 3). Similar to the previous model, WC increased by 4.259 centimeters for every SD unit increase in ln Ocdd (Table 3). Although the WC interaction was positive for ln hpcdd, male WC tended to increase by 2.305 centimeters whereas females decreased by 2.504 centimeters for every SD unit increase ln hpcdd (Table 4). For the ln DDT interaction, male BMI decreased by 0.852 while female BMI increased by 2.041 for every SD unit increase in ln DDT (Table 4). The joint test p-value for simultaneous testing for the interactions in the interaction WC model is <0.00005.

Lastly, the interaction WC model controlling for BMI accounted for 88% of the WC variance and showed significant associations for ln hpcdd (p = 0.0060) with a significant gender by ln DDT interaction (p = 0.0056) (Table 3). Not surprisingly, age and BMI were both positively associated with WC. However, as in the previous model, WC decreased by 0.452 cm for every SD unit increase in ln hpcdd (Table 3). The significant interaction shows that male WC decreases by 0.120 cm whereas female WC increases by 0.111 cm for every SD unit increase in ln DDT (Table 4). The joint test p-value for simultaneously testing for the interactions in the interaction WC model controlling for BMI is 0.0050.

### 3.3. Secondary (Sensitivity) Analysis

#### 3.3.1. Additive Model

Results of the additive regression models for participants with detectable POPs are presented in Table 5.

The additive BMI model accounted for 13% of the BMI variance and showed significant associations for ethnicity (p = 0.0450), age (p < 0.00005), age squared (p < 0.00005), ln trans-nonachlor (p = 0.0483), and ln DDT (p = 0.0340) (Table 5). With the exception of ln trans-nonachlor, all of the effects were the same and in the same direction as in the primary additive BMI model. Here BMI decreases by 1.514 for every SD unit increase in ln trans-nonachlor (Table 5). The joint test for simultaneously testing the POPs was significant with *p* = 0.0017. The model accounted for 2.8% of BMI.

For the additive WC model, significant associations, and in the same direction, for gender (p < 0.00005), age (p < 0.00005), and age squared (p < 0.00005) appeared (Table 5). However, unlike the additive WC model for all participants, ln Ocdd was not significant. The model accounted for 20% of the variance (Table 5). The joint test for simultaneously testing for the POPs was significant with (p = 0.0129).

For the additive WC model controlling for BMI, the model accounted for 87% of the WC variance (Table 5). With the exception of ln hpcdd, the same significant associations in the same direction were observed as with the model with all participants. Here, ln hpcdd was marginally significant (p = 0.0527), but it was still in the same direction. The joint test simultaneously testing all POPs was not significant.

#### 3.3.2. Interaction Models

Table 6 displays the results of the interaction regression models for participants with detectable POPs, and Table 7 displays the interaction regression equations for males and females. The interaction BMI model accounted for 15% of the variance and showed the same significant associations for age (p < 0.00005), age squared (p < 0.00005), ln Ocdd (p = 0.0277), and ln DDT (p = 0.0340) (Table 6). However, here there were no significant gender interactions with ln hpcdd or ln DDT. Instead, there was a gender interaction with ln oxychlordane (p = 0.0204) (Table 6). Further, inspection of the interactions through Table 7 shows that male BMI increases by 3.483 and female BMI decreases by 0.323 for every SD unit increase in ln oxychlordane (Table 7). The simultaneous test for the gender by POPs interactions was significant with p = 0.0121.

The interaction WC model accounted for 23% of the variance and showed significant associations for gender (p = 0.0112), age (p < 0.00005), age squared (p < 0.00005), ln Ocdd (p = 0.0123), and ln DDT (p = 0.0184) (Table 6). The significant associations were in the same direction as the interaction WC model with all participants. The model also showed significant gender interactions with ln oxychlordane (p = 0.0119) and DDT (p = 0.0409) (Table 6). The gender by ln hpcdd was not significant in this model. The gender by ln oxychlordane interaction was in the same direction as the primary WC model. Table 7 shows that for the gender by ln Ocdd interaction male WC decreases by 0.398 and female BMI increases by 6.054 for every SD unit increase in ln Ocdd. The gender by DDT interaction indicates that male WC decreases by 0.193 and female WC increases by 2.260 for every SD unit increase in DDT. The joint test for simultaneously testing for the gender by POPs interactions was significant with (p = 0.0011).

Finally, the interaction WC model controlling for BMI accounted for 88% of the WC variance with significant main associations for ethnicity(p = 0.0299), age (p = 0.0105), BMI (p < 0.00005), BMI squared (p = 0.0211), and ln hpcdd (p = 0.0214) (Table 6). The ethnicity main effect was the only difference in main effects between the current model and primary WC conditional on BMI model. Even so, it appears that African Americans have 2.05 lower WC than other races. With regard to interactions, there are significant gender-by-ln hpcdd (p = 0.0283), and gender-by-ln oxychlordane (p = 0.0231) interactions (Table 6). The gender-by-ln DDT interaction was not significant here. Inspection of Table 7 shows that male BMI increases by 0.129 and female BMI decreases by 0.917 for every SD unit increase in ln hpcdd. However, male BMI increases by 1.577 whereas female BMI decreases by 1.793 for every SD unit increase in ln oxychlordane (Table 7). The joint test for simultaneously testing the gender-by- POPs interactions was significant with p = 0.0012.

#### 3.3.3. Comparison of all participants and participants with detectable POPs models:

Consistent findings for the additive models with all participant and participants with detectable POPs were as follows: The additive BMI models showed consistent associations for ethnicity, age, age squared, and ln DDT. For the additive WC model, the consistent associations were for gender, age, and ln Ocdd. Lastly, the additive WC model controlling for BMI show consistent finding for gender, age, BMI, and BMI squared.

Consistent findings for the interaction models for all participants and participants with detectable POPs were as follows: The interaction BMI model showed consistent associations for age, age squared, and ln Ocdd. The interaction WC model showed consistent associations for age, ln Ocdd, and ln DDT. In addition, it had consistent gender by ln oxychlordane and ln DDT interactions. Lastly, the interaction WC model controlling for BMI had consistent associations for age, BMI, BMI squared, and ln hpcdd.

Lastly, the additive and interaction models were estimated with all participants ages 19 and up (results not shown but available upon request). Results of these analyses were consistent with the results of the equivalent models with all participants. Specifically, all parameter estimates were in the same directions and those that were significant (or not significant) remained significant (or not significant). The one exception occurred with the interaction model of waist circumference controlling for BMI. In this model the parameter estimate for European Americans went from non significant to significant and the parameter estimate for age went from significant to non significant.

## 4. Conclusions

Consistent effects were found for Ocdd and/or DDT with BMI. In addition, a relatively consistent association between WC and hpcdd controlling for BMI was found. To the extent that our associations can be speculated to represent causation, on average, one of the toxic effects of these chemicals appears to be weight gain. Unlike the well-known weight loss resulting from high exposure to POPs, this weight gain may occur at much lower levels of exposure, levels which fail to make animals or humans obviously ill [21]. A couple opposite gender effects was also identified. Oxychlordane is associated with BMI increases in males but a BMI decreases in females (Tables 6 and 7). Conversely, DTT is associated with WC decreases in males but WC increases in females (Table 4). A speculation, which needs further validation, is that this might have to do with hormonally-directed differences in fat storage in men *versus* women. Men tend to be “apples” and store their fat in the waist, whereas females tend to be “pears” and store their fat in their hips. POPs might be affecting this process.

Because of their previous extensive usage as pesticides, their inherent structural stability, their persistence in body systems and their ability to concentrate in animals that are higher up on the food chain, many POPs are currently present in human fat in relatively high levels. Much of any chemical-induced weight gain may come from increases in the overall proportion of body fat. In one animal study, the pesticide dieldrin more than doubled the total body-fat content of treated mice [22]. Another study showed that a pesticide, commonly known as lindane, induced obesity in animals [23]. In yet another study, the overall weight gain effect of another pesticide, hexachlorobenzene, appeared to be so powerful that a group of treated animals still managed to gain significantly more weight despite the fact that their food intake was cut by 50% relative to untreated controls that were on full food rations [24].

Persistent organic pollutants, synthetic and industrial chemicals, appear to cause weight gain by interfering with most of the different elements that comprise the human weight control system. In particular, these chemicals have been shown to disrupt major weight controlling hormones, such as thyroid hormones, estrogens, testosterone, corticosteroids, insulin, growth hormone, and leptin [25] and to alter levels of, and sensitivity to, neurotransmitters (in particular dopamine, noradrenaline, and serotonin [26]. They interfere with many metabolic processes and cause widespread damage to body tissues and cardiovascular disease [27]. This interference may result in changes in appetite, in food efficiency, and in fat, carbohydrate, and protein metabolism. The desire, and ability, to exercise also may be affected. These changes have been thought to be responsible for increases in body weight [21,23,28,29].

*What are the contributions of POPs to pre-diabetes and diabetes disorders?* Lee and colleagues have recently shown a dose-response relation between serum concentrations of POPs and metabolic syndrome [17], insulin resistance [18], and diabetes [19]. They claimed that the expected association between obesity and diabetes was absent with people with low concentrations of POPs [19]. However, Porta argues that the adjustment for BMI and WC in the published studies might be an over adjustment, because dietary fats are a major source of POPs [36].

These investigators determined that POPs have a much greater association with these diseases in obese people compared with non-obese people. They hypothesize that the toxicity of POPs related to the risk of metabolic syndrome, insulin resistance, and diabetes substantially increases as people get more obese [17]. However, another possibility is that POPs, in addition to their contribution to metabolic syndrome, insulin resistance, and diabetes, might also directly contribute to obesity. The complex interactions among POPs, obesity, and diabetes-related disorders make the determination of the contribution of a single component difficult. Long-term longitudinal cohort studies with repeated measurements of BMI, WC, and POPs levels and continued model organism experimentation could help solve this problem.

*Are environmental chemicals responsible for the obesity epidemic?* Keith and colleagues *coined* the phrase “The Big Two” to refer to two commonly presumed causes of the obesity epidemic, namely food marketing practices and institutionally-driven reductions in physical activity [30]. The Big Two are likely contributors, but there are several other likely contributors, such as POPs [30]. The particular POPs studied herein were chosen in this study because they are present in over 80% of the NHANES population. However, the concentrations of some POPs in general have been decreasing in the past two decades in the U.S. population because of stricter regulation [31], whereas obesity has been increasing during this same period. According to NHANES data, obesity (*i.e*., BMI ≥ 30) has increased from almost 30% of the adult population in 1980 to over 60% of the population in 2000 [31]. How then can we explain the strong dose-response relation between serum concentrations of POPs and obesity? A possibility is that the effects of the POPs were on neonates 20 years ago, and that this is only now becoming manifest in the adult population. This theory is called the “developmental origins of health and disease” (DOHaD), or the “Barker hypothesis,” because it was first proposed by Barker and colleagues [32].

An excellent example of DOHaD that is related to POPs and obesity is recent work done by Retha Newbold and colleagues [16,33]. In pioneering work started in the 1970s, Dr. Newbold and colleagues showed that the potent estrogenic compound diethylstilbesterol (DES) can increase the incidence of uterine cancer and testicular atrophy in the children and grandchildren of pregnant mothers given this compound, which was used in humans to treat morning sickness [34,35]. In recent studies, they showed that low doses of DES (0.001 mg/kg) given every day for the first 5 days after birth can induce obesity in post-pubertal mice [16,33]. Whether a similar phenomenon occurs in humans will be the subject of future investigations.

## Figures and Tables

**Table 1:** Descriptive statistics for detectable persistent organic pollutants.

	BMI			Waist Circumference		

	n	Mean	95% CI	n	Mean	95% CI

Gender						
Male	1,140	27.75 (0.28)	27.17, 28.33	1,133	98.53 (0.72)	97.07, 100.00
Female	1,324	27.55 (0.26)	27.01, 28.09	1,315	91.82 (0.80)	90.19, 93.46
Ethnicity						
Mexican American	758	27.77 (0.27)	27.22, 28.33	749	92.94 (0.61)	91.69, 94.19
Non-Hispanic Black	112	28.09 (0.83)	26.39, 29.79	112	95.19 (2.67)	89.72, 100.66
Non-Hispanic White	1,022	27.47 (0.28)	26.90, 28.04	1,026	95.49 (0.80)	93.85, 97.14
Other Hispanic	485	28.91 (0.37)	28.15, 29.68	475	94.92 (1.03)	92.82, 97.02
Other Race	87	26.71 (0.68)	25.32, 28.10	86	90.34 (1.95)	86.34, 94.33
Age						
06–18 yrs	504	23.17 (0.32)	22.52, 23.82	500	80.52 (0.81)	78.86, 82.18
19–28 yrs	397	26.71 (0.44)	25.81, 27.60	391	90.60 (1.23)	88.08, 93.12
29–39 yrs	369	27.84 (0.49)	26.84, 28.84	367	93.64 (1.45)	90.68, 96.60
40+ yrs	1,194	28.42 (0.24)	27.92, 28.91	1,190	98.49 (0.70)	97.07, 99.92

Note. Numbers in parentheses are standard errors of the mean.

**Table 2:** Additive regression models for all participants.

	BMI		WC		WC|BMI	

Variable	Beta	p-value	Beta	p-value	Beta	p-value

Intercept	13.26 (2.83)	0.0001	51.75 (7.43)	<0.00005	7.46 (4.15)	0.0827
Mexican American	0.69 (0.86)	0.4263	1.04 (2.37)	0.6629	−0.58 (0.77)	0.4590
Other Hispanic	1.27 (1.08)	0.2518	3.42 (3.30)	0.33088	−0.12 (1.00)	0.9028
White	0.38 (0.82)	0.6442	2.16 (2.43)	0.3815	1.56 (0.92)	0.0982
African American	1.89 (0.91)	0.0466	2.93 (2.46)	0.2431	−1.28 (0.90)	0.1688
Male	0.63 (0.39)	0.1192	8.01 (1.05)	<0.00005	6.40 (0.37)	<.00005
Age	0.33 (0.04)	<0.00005	0.89 (0.10)	<0.00005	0.11 (0.04)	0.0056
Age-2	−0.00 (0.005)	<0.00005	−0.01 (0.005)	<0.00005	0.00 (0.005)	0.6518
BMI	.	.	.	.	3.33 (0.20)	<0.00005
BMI-2	.	.	.	.	−0.02 (0.005)	<0.00005
ln(hpcdd)	0.44 (0.29)	0.1370	0.24 (0.74)	0.7476	−0.68 (0.24)	0.0091
ln(Ocdd)	0.83 (0.30)	0.0099	2.31 (0.76)	0.0052	0.31 (0.25)	0.2184
ln(oxychlordane)	0.58 (0.64)	0.3716	1.59 (1.63)	0.3359	0.16 (0.48)	0.7404
ln(trans-nonachlor)	−0.74 (0.60)	0.2258	−1.72 (1.52)	0.2697	0.04 (0.33)	0.9047
ln(DDT)	0.48 (0.22)	0.0353	0.81 (0.52)	0.1283	−0.17 (0.22)	0.4610

Joint Test p-value	0.0001		0.0008		0.0798	
R-Squared	0.0793		0.1579		0.8762	
R-Squared (No POPs)	0.0591		0.1449		0.8756	
N	2,464		2,448		2,428	

Note. WC stands for waist circumference. Numbers in parentheses are standard errors. Joint Test refers to the simultaneous test of all POPs.

**Table 3:** Gender Interaction Regression Models for All Participants.

	BMI		WC		WC|BMI	

Variable	Beta	p-value	Beta	p-value	Beta	p-value

Intercept	13.46 (3.19)	0.0002	52.91 (9.37)	<0.00005	8.58 (5.19)	0.1090
Mexican American	0.95 (0.85)	0.2744	1.78 (2.33)	0.4514	−0.39 (0.77)	0.6143
Other Hispanic	1.43 (1.04)	0.1792	3.84 (3.15)	0.2316	−0.01 (1.00)	0.9921
European American	0.54 (0.81)	0.5068	2.60 (2.35)	0.2787	1.67 (0.89)	0.0711
African American	2.08 (0.90)	0.0286	3.39 (2.39)	0.1674	−1.15 (0.88)	0.2019
Male	−0.97 (5.27)	0.8550	2.41 (12.12)	0.8439	3.90 (4.36)	0.3782
Age	0.31 (0.04)	<0.00005	0.86 (0.10)	<0.00005	0.10 (0.04)	0.0084
Age-2	−0.003 (0.005)	<0.00005	−0.01 (.005)	<0.00005	0.00 (0.005)	0.5574
BMI	.	.	.	.	3.31 (0.21)	<0.00005
BMI-2	.	.	.	.	−0.02 (0.005)	0.0001
ln(hpcdd)	−0.37 (0.46)	0.4172	−2.00 (1.25)	0.1211	−1.16 (0.39)	0.0060
ln(Ocdd)	1.23 (0.44)	0.0091	3.38 (1.26)	0.0120	0.50 (0.46)	0.2839
ln(oxychlordane)	−0.21 (0.88)	0.8135	−0.51 (2.26)	0.8235	−0.29 (0.78)	0.7081
ln(trans-nonachlor)	−0.55 (0.80)	0.4971	−1.49 (2.01)	0.4650	0.05 (0.65)	0.9414
ln(DDT)	1.08 (0.32)	0.0020	2.73 (0.75)	0.0010	0.34 (0.33)	0.3147
male* ln(hpcdd)	1.77 (0.55)	0.0033	4.85 (1.63)	0.0059	1.03 (0.67)	0.1384
male* ln(Ocdd)	−0.69 (0.67)	0.3103	−1.82 (1.75)	0.3087	−0.26 (0.69)	0.7141
male* ln(oxychlordane)	1.68 (0.96)	0.0896	4.59 (2.46)	0.0718	1.03 (0.82)	0.2229
male* ln(trans-nonachlor)	−0.47 (0.80)	0.5611	−0.78 (2.05)	0.7054	−0.12 (0.87)	0.8908
male* ln(DDT)	−1.24 (0.42)	0.0060	−3.97 (0.97)	0.0003	−1.04 (0.35)	0.0056

Joint Test p-value	0.0005		<.00005		0.0050	
R-Squared	0.0953		0.1793		0.8775	
N	2464		2448		2428	

Note. WC stands for waist circumference. Numbers in parentheses are standard errors. Joint Test refers to the simultaneous test of all two-way gender by POPs interactions.

**Table 4:** Gender Interaction Equations for All Participants.

	BMI		WC		WC|BMI	

Variable	Male	Female	Male	Female	Male	Female

Intercept	12.493 (4.493)	13.464 (3.194)	55.315 (9.411)	52.907 (9.369)	12.481 (4.146)	8.578 (5.188)
Mexican American	0.948 (0.851)	0.948 (0.851)	17.780 (2.331)	1.779 (2.331)	−0.390 (0.766)	−0.390 (0.766)
Other Hispanic	1.426 (1.036)	1.426 (1.036)	3.844 (3.146)	3.844 (3.146)	−0.010 (1.000)	−0.010 (1.000)
European American	0.541 (0.806)	0.541 (0.806)	2.596 (2.352)	2.596 (2.352)	1.672 (0.892)	1.672 (0.892)
African American	2.081 (0.903)	2.081 (0.903)	3.391 (2.395)	3.391 (2.395)	−1.145 (0.877)	−1.145 (0.877)
Age	0.314 (0.041)	0.314 (0.041)	0.858 (0.099)	0.858 (0.099)	0.101 (0.036)	0.101 (0.036)
Age-2	−0.003 (0.0004)	−0.003 (0.0004)	−0.007 (0.001)	−0.007 (0.001)	0.0002 (0.0004)	0.0002 (0.0004)
BMI	.	.	.	.	3.311 (0.210)	3.311 (0.210)
BMI-2	.	.	.	.	−0.0165 (0.003)	−0.0165 (0.003)
ln(hpcdd)	1.391 (0.297)	−0.375 (0.455)	2.849 (0.809)	−2.00 (1.252)	−0.132 (0.434)	−1.161 (0.392)
ln(Ocdd)	0.540 (0.483)	1.230 (0.440)	1.559 (1.091)	3.77 (1.259)	0.243 (0.391)	0.499 (0.457)
ln(oxychlordane)	1.471 (0.684)	−0.209 (0.878)	4.084 (1.651)	−0.508 (2.258)	0.732 (0.440)	−0.293 (0.776)
ln(trans-nonachlor)	−1.02 (0.630)	−0.552 (0.802)	−2.270 (1.635)	−1.487 (2.008)	−0.072 (0.432)	0.048 (0.647)
ln(DDT)	−0.163 (0.307)	1.081 (0.318)	−1.242 (0.686)	2.728 (0.748)	−0.704 (0.170)	0.337 (0.329)

Note. WC stands for waist circumference. Numbers in parentheses are standard errors.

**Table 5:** Additive Regression Models for Participants with Detectable Persistent Organic Pollutants.

	BMI		WC		WC|BMI	

Variable	Beta	p-value	Beta	p-value	Beta	p-value

Intercept	12.54 (3.61)	0.0016	53.02 (8.35)	<0.00005	11.45 (6.11)	0.0711
Mexican American	0.21 (0.96)	0.8309	−0.62 (2.72)	0.8196	−0.99 (0.93)	0.2926
Other Hispanic	−0.13 (1.25)	0.9146	−1.63 (3.38)	0.6329	−1.11 (1.11)	0.3234
European American	0.45 (1.00)	0.6523	1.11 (2.84)	0.6990	0.56 (1.01)	0.5870
African American	2.43 (1.16)	0.0450	3.26 (3.11)	0.3039	−1.99 (0.92)	0.0388
Male	0.35 (0.36)	0.3361	6.96 (1.06)	<0.00005	5.85 (0.45)	<0.00005
Age	0.44 (0.05)	<0.00005	1.15 (0.12)	<0.00005	0.12 (0.04)	0.0045
Age-2	−0.00 (0.005)	<0.00005	−0.01 (0.005)	<0.00005	0.00 (0.005)	0.8474
BMI	.	.	.	.	3.25 (0.36)	<0.00005
BMI-2	.	.	.	.	−0.02 (0.01)	0.0167
ln(hpcdd)	0.62 (0.43)	0.1554	0.51 (1.17)	0.6677	−0.77 (0.38)	0.0527
ln(Ocdd)	0.62 (0.40)	0.1344	1.69 (0.94)	0.0843	0.13 (0.30)	0.6732
ln(oxychlordane)	1.56 (0.91)	0.0979	3.91 (2.41)	0.1147	0.08 (1.06)	0.9401
ln(trans-nonachlor)	−1.76 (0.86)	0.0483	−3.94 (2.28)	0.0940	0.26 (0.68)	0.7114
ln(DDT)	0.55 (0.25)	0.0340	0.98 (0.53)	0.0736	−0.14 (0.24)	0.5575

Joint Test p-value	0.0017		0.0129		0.2207	
R-Squared	0.1310		0.2030		0.8736	
R-Squared (No POPs)	0.1026		0.1881		0.8727	
N	1,727		1,709		1,696	

Note. WC stands for waist circumference. Numbers in parentheses are standard errors. Joint Test refers to the simultaneous test of all POPs.

**Table 6:** Gender Interaction Regression Models for Detectable Persistent Organic Pollutants.

	BMI		WC		WC|BMI	

Variable	Beta	p-value	Beta	p-value	Beta	p-value

Intercept	6.53 (4.57)	0.1637	39.17 (10.98)	0.0013	10.60 (7.06)	0.1440
Mexican American	0.21 (0.94)	0.8204	−0.54 (2.60)	0.8371	−0.94 (0.94)	0.3258
Other Hispanic	−0.23 (1.24)	0.8539	−1.85 (3.30)	0.5788	−0.10 (1.12)	0.3329
European American	0.38 (0.95)	0.6932	0.91 (2.68)	0.7380	0.49 (1.01)	0.6314
African American	2.28 (1.14)	0.0550	2.81 (2.99)	0.3553	−2.06 (0.90)	0.0299
Male	12.35 (5.50)	0.3250	34.31 (12.66)	0.0112	8.37 (5.68)	0.1513
Age	0.42 (0.05)	<0.00005	1.10 (0.12)	<0.00005	0.11 (0.04)	0.0105
Age-2	−0.00 (0.005)	<0.00005	−0.01 (0.005)	<0.00005	0.00 (0.005)	0.6216
BMI	.	.	.	.	3.21 (0.37)	<0.00005
BMI-2	.	.	.	.	−0.02 (0.01)	0.0211
ln(hpcdd)	0.18 (0.65)	0.7877	−1.18 (1.87)	0.5323	−1.49 (0.61)	0.0214
ln(Ocdd)	1.42 (0.61)	0.0277	3.84 (1.58)	0.0213	0.58 (0.48)	0.2329
ln(oxychlordane)	−0.24 (1.37)	0.8638	−1.04 (3.64)	0.7770	−1.26 (1.43)	0.3847
ln(trans-nonachlor)	−1.21 (1.18)	0.3148	−2.47 (3.21)	0.4480	0.88 (1.18)	0.4614
ln(DDT)	0.98 (0.44)	0.0340	2.38 (0.95)	0.0184	0.17 (0.37)	0.6565
male* ln(hpcdd)	0.96 (0.76)	0.2172	3.83 (2.34)	0.1129	1.80 (0.78)	0.0283
male* ln(Ocdd)	−1.55 (0.80)	0.0637	−4.18 (2.21)	0.0681	−0.89 (0.85)	0.3067
male* ln(oxychlordane)	3.74 (1.52)	0.0204	10.43 (3.89)	0.0119	2.94 (1.23)	0.0231
male* ln(trans-nonachlor)	−1.34 (1.06)	0.3316	−3.68 (3.51)	0.3037	−1.55 (1.30)	0.2423
male* ln(DDT)	−0.83 (0.56)	0.1503	−2.65 (1.24)	0.0409	−0.53 (0.43)	0.2285

Joint Test p-value	0.0121		0.0011		0.0012	
R-Squared	0.1480		0.2259		0.8757	
N	1727		1709		1696	

Note. WC stands for waist circumference. Numbers in parentheses are standard errors. Joint Test refers to the simultaneous test of all two-way gender by POPs interactions.

**Table 7:** Gender interaction equations for detectable persistent organic pollutants.

	BMI		WC		WC|BMI	

Variable	Male	Female	Male	Female	Male	Female

Intercept	18.877 (4.107)	6.530 (4.569)	73.476 (8.789)	39.167 (10.985)	18.966 (6.742)	10.596 (7.056)
Mexican American	0.214 (0.936)	0.214 (0.936)	−0.540 (2.602)	−0.540 (2.602)	−0.941 (0.942)	−0.941 (0.942)
Other Hispanic	−0.230 (1.237)	−0.230 (1.237)	−1.850 (3.295)	−1.850 (3.295)	−1.105 (1.122)	−1.105 (1.122)
European American	0.379 (0.951)	0.379 (0.951)	0.905 (2.680)	0.905 (2.680)	0.488 (1.006)	0.486 (1.006)
African American	2.282 (1.141)	2.282 (1.141)	2.810 (2.991)	2.810 (2.991)	−2.061 (0.902)	−2.061 (0.902)
Age	0.423 (0.050)	0.423 (0.050)	1.095 (0.118)	1.095 (0.118)	0.002 (0.041)	0.112 (0.041)
Age-2	−0.004 (0.0005)	−0.004 (0.0005)	−0.009 (0.001)	−0.009 (0.001)	0.0002 (0.0005)	0.0002 (0.0005)
BMI	.	.	.	.	3.205 (0.369)	3.205 (0.369)
BMI-2	.	.	.	.	−0.0152 (0.006)	−0.015 (0.006)
ln(hpcdd)	1.134 (0.423)	0.176 (0.647)	2.654 (1.207)	−1.179 (1.865)	0.305 (0.423)	−1.493 (0.614)
ln(Ocdd)	−0.129 (0.481)	1.418 (0.611)	−0.342 (1.164)	3.839 (1.577)	−0.302 (0.588)	0.585 (0.480)
ln(oxychlordane)	3.504 (0.994)	−0.236 (1.367)	9.392 (2.443)	−1.042 (3.645)	1.683 (0.937)	−1.258 (1.425)
ln(trans-nonachlor)	−2.550 (0.991)	−1.212 (1.185)	−6.152 (2.439)	−2.473 (3.215)	−0.665 (0.581)	0.880 (1.179)
ln(DDT)	0.154 (0.324)	0.982 (0.441)	−0.278 (0.699)	2.377 (0.951)	−0.366 (0.215)	0.165 (0.368)

Note. WC stands for waist circumference. Numbers in parentheses are standard errors.
